# The Role of Race and Poverty in Access to Foods That Enable Individuals to Adhere to Dietary Guidelines

**Published:** 2006-06-15

**Authors:** Elizabeth A Baker, Mario Schootman, Ellen Barnidge, Cheryl Kelly

**Affiliations:** Saint Louis University School of Public Health, Salus Center; Department of Medicine and Pediatrics, Washington University School of Medicine, St Louis, Mo; Saint Louis University School of Public Health, St Louis, Mo; Saint Louis University School of Public Health, St Louis, Mo

## Abstract

**Introduction:**

The increase in obesity and disparities in obesity and related chronic diseases across racial and ethnic and income groups have led researchers to focus on the social and environmental factors that influence dietary intake. The question guiding the current study was whether all communities have equal access to foods that enable individuals to make healthy dietary choices.

**Methods:**

We conducted audits of community supermarkets and fast food restaurants to assess location and availability of food choices that enable individuals to meet the dietary guidelines established by the U.S. Department of Agriculture (e.g., fruit and vegetable consumption, low-fat options). We used 2000 census data to assess the racial distribution and the percentage of individuals living below the federal poverty level in a defined area of St Louis, Mo. Spatial clustering of supermarkets and fast food restaurants was determined using a spatial scan statistic.

**Results:**

The spatial distribution of fast food restaurants and supermarkets that provide options for meeting recommended dietary intake differed according to racial distribution and poverty rates. Mixed-race or white high-poverty areas and all African American areas (regardless of income) were less likely than predominantly white higher-income communities to have access to foods that enable individuals to make healthy choices.

**Conclusion:**

Without access to healthy food choices, individuals cannot make positive changes to their diets. If certain eating behaviors are required to reduce chronic disease and promote health, then some communities will continue to have disparities in critical health outcomes unless we increase access to healthy food.

## Introduction

Obesity is one of the leading health concerns in the United States; approximately 65% of American adults are overweight or obese (1,2). Previous studies have found that poor nutrition and physical inactivity are key risk factors in the development of obesity (3-7).

Recent findings indicate that rates of obesity are higher among some racial and ethnic minority groups as well as among lower-income groups. For example, 38% of African Americans are obese, compared with 27% of Hispanics, 37% of Native Americans, and 21% of the entire U.S. population (8). Data also suggest that lower-income groups have higher rates of obesity than their higher-income counterparts; this trend is particularly apparent among women (9,10). In addition, there are disparities in rates of chronic diseases related to obesity. For example, in 2000 the death rate from heart disease among African Americans was 29% higher than among non-Hispanic whites (11). Similarly, African Americans are twice as likely as non-Hispanic whites to be diagnosed with diabetes (12).

Previous studies indicate that the etiology of obesity is multifactorial. Although much of the initial work on obesity focused on individual and interpersonal factors, public health practitioners are becoming increasingly interested in the environmental and broader social determinants (e.g., race and ethnicity, poverty) of obesity (13-23).

Several studies have been conducted to examine environmental influences, such as the association between the location of food outlets and the consumption of various types of food. For example, Moreland et al found that more fruits and vegetables were consumed in areas with more supermarkets (17). Other studies have examined the extent to which the location of food outlets is associated with various area-level factors such as race, ethnicity, or income. These studies have found that lower income and predominantly African American neighborhoods have fewer supermarkets (or longer distances to markets) but more fast food restaurants (18,19,21).

Other researchers have suggested that the ability to make healthy choices is influenced not only by the location of the food outlet but also by the selection of items in the outlet (24). For example, Cheadle et al examined the selection of a broad range of foods within supermarkets and found that increased selection of low-fat and high-fiber foods was associated with healthier dietary consumption; they did not, however, investigate whether selection differed by area-level factors such as race and ethnicity or poverty rate (20).  

Our study reports on the association between location and selection of foods that enable individuals to make healthy choices and racial distribution and poverty rates. We used direct observations through audit tools as well as existing databases (the Web sites of fast food restaurant corporations and U.S. census data). Our analysis techniques allowed us to examine not only the variance between these areas but also whether the differences were significantly more or less than would be expected based on population density.

## Methods

We audited supermarkets and fast food restaurants for the availability of healthy food choices. The data were analyzed using a geographic information system (GIS) and geographic clustering software. We obtained information on racial and ethnic distribution and percentage of the population living in poverty from the 2000 U.S. census. We determined whether the area-level characteristics were associated with clustering of supermarkets and fast food restaurants.

### Study area 

The study area consisted of the city of St Louis, Mo, and the eastern part of St Louis County, Missouri, the area between the Missouri River on the east and Interstate 270 (the outer belt of the St Louis area) on the west. This area is considered by many residents to comprise the urban area of St Louis. This area includes 233 square miles, 220 census tracts, and 912,323 people (25).

### Development of audit tools 

The audit tools developed for this study build upon previous work (20) and reflect current dietary intake guidelines established by the U.S. Department of Agriculture (USDA) (26). The recommendations include eating a variety of fruits and vegetables each day and selecting from all five vegetable subgroups (dark green, orange, legumes, starchy vegetables, and other vegetables). In addition, the recommendations suggest eating lean, low-fat, or fat-free meat, poultry, and dairy products. The audit tools were designed to assess the extent to which the environment — supermarkets and fast food restaurants — provided individuals with a selection of foods that would enable them to follow these recommendations.

**Supermarket audit tool**

The supermarket audits were structured to determine the extent to which the selection of foods available in each supermarket enabled individuals to meet USDA recommendations. The fruit and vegetable section of the audit tool was created as a checklist that included each item identified by the USDA's Continuing Survey of Food Intakes by Individuals (CSFII) as currently being consumed by adults living in urban midwestern cities (78 total fruits and vegetables). The checklist provided a place for the auditor to indicate whether each item was available in a fresh, frozen, or canned form in each store.

We used the USDA's Agriculture Handbook 8 (27) to develop the audit tool for assessing the availability of lean, low-fat, and fat-free meat, poultry, and dairy products. Similar to the fruit and vegetable section of the audit tool, this section was created as a checklist for the auditor to indicate whether each type of lean beef, skinless chicken, or low-fat or reduced-fat cheese was available in each store. The audit tool also allowed the recorder to identify the availability of fat-free, 1/2%, 1%, 1 1/2%, 2%, and whole milk.

**Fast food restaurant audit tool**

The fast food restaurant audit tool assessed the extent to which the menu options at each fast food restaurant provided the opportunity for individuals to meet the recommended dietary intake based on the availability and preparation of the foods (e.g., broiled or baked rather than fried). *Fast food restaurants* were defined as restaurants where customers place orders at the counter (i.e., with no waitress or waiter available for service). Twenty-six major fast food restaurant chains were audited including hamburger, sandwich, Mexican, chicken, and pizza chains. All fast food restaurants in this study provided menus through a corporate Web site. A checklist was created based on the menu of each chain (24). Audit tools were designed for each restaurant chain so that all items included on the corporate menu that would enable customers to meet one of the recommended eating patterns was placed on a checklist. For example, the items on the corporate menu (and thus the checklist) that were identified as providing the opportunity to eat fruits and vegetables may have included a garden salad, a chef salad, or a taco salad, depending on the chain. Each auditor used the corporation-specific checklist to assess which of the food options that met the recommended dietary guidelines were carried by local branches or franchises.

**Conducting audits**

Between 2003 and 2004, audits were conducted in person at all stores that were identified by the 2000 business census as either supermarkets or major-chain grocery stores and had addresses that were geographically located within the study area (N = 81). Each store was audited by two research staff: one observer (who visually noted all the items) and one recorder (who recorded the items on a standard data sheet). Each auditor participated in a half-day training session and followed an auditor for another half day. Using the 78-item fruits and vegetable checklist, the auditor recorded whether each store carried that fruit or vegetable and whether it was available fresh, frozen, or canned. Similarly, each auditor looked for all available meat, poultry, and dairy options. The auditors checked the checklist for each item the supermarket carried; they did not count the number of each item the store had. (For example, they noted whether there were peaches but not the number of peaches.) This process was chosen because variations in the actual number of peaches (or other items) might reflect purchasing and stocking patterns within the store rather than the availability of an item.

A two-stage process was used to assess the selection of options that met dietary intake recommendations at local fast food restaurants. The first phase entailed stratifying the study area into census tracts by three racial and three poverty groups. Each fast food restaurant was placed within a stratum based on its location. A random sample of two of each type of fast food chain within each census tract was then audited by telephone. These audits were conducted by auditors who participated in a half-day training session. Once the manager or supervisor of the fast food restaurant agreed to an audit, the auditor went through the checklist of items based on the corporate menu and asked the manager or supervisor to indicate whether each item was available at the branch or franchise.

The data were reviewed after 130 fast food restaurants, or approximately half the sample, had been audited. It was then determined that there were few differences in the availability of healthier options within chains, regardless of geographic location or stratum (race or income). Overall, the variance between restaurants within the same chain was less than 5% for most chains (ranging from 0% to 5%). The lack of variability from our calls, along with the possibility of respondent bias, resulted in a decision to score each fast food restaurant based on its corporate menu. Although the restaurants may have varied more than we ascertained through our sampling and may not have had all of the items from the corporate menu, this method allowed us to give each restaurant its best possible rating.

### Statistical analysis 

**Availability of healthy choices**

A composite score was created for each supermarket by combining the observed number of different fruits and vegetables available and lean, low-fat, and fat-free meat, poultry, and dairy options. Using the composite score, *z* scores were calculated for each supermarket. The *z* scores, based on the mean availability and standard deviation of all items, allowed comparisons among supermarkets. Tertiles were created to indicate high, medium, or low availability of fruits and vegetables and low-fat options based on the distribution of *z* scores.

Each fast food restaurant audited also received a composite score based on the total number of items available that met dietary guidelines. The *z* scores were calculated for each restaurant. Based on the distribution of *z* scores, ratings were divided into tertiles and labeled high, medium, or low potential for meeting dietary intake recommendations.

**2000 census data**

To determine racial distribution and poverty rates in the study area, we used 2000 U.S. census data at the census-tract level. Only two racial groups were considered because, according to the U.S. Census Bureau, 95% of the population residing in the study area self-identifies as white or African American. Census tracts were identified as primarily African American if 75% or more of the population in the area self-identified as African American or as primarily white if 75% or more of the population in the area self-identified as white. All other areas were identified as mixed.

The percentage of the population living below the U.S. federal poverty level was measured using 2000 U.S. census data. The poverty rate is a measure that seems to be robust across various diseases and levels of geography; it has a link to possible policy implications; and it is comparable over time (28,29). We grouped poverty rate into three levels: less than 10% of the population living in poverty, 10% to 19.9% of the population living in poverty, and 20% or more of the population living in poverty.

**Address matching and spatial clustering**

The street addresses of the fast food restaurants identified were converted to approximate geographic locations and assigned a latitude and longitude. First, all addresses were preprocessed using ZP4 (Semaphore Corp, Pismo Beach, Calif) and then address-matched using ArcView 3.2 (ESRI, Redlands, Calif) with the Redistricting Census 2000 TIGER/Line as the reference files. Unmatched or questionably matched addresses (scoring lower than 85) were recoded using the Internet-based EZ-Locate system (Tele Atlas North America, Inc, Lebanon, NH). Of the 32 unmatched addresses, one was matched to the centroid of the ZIP code, one was matched to the centroid of the ZIP+2 code, one was a near match, and the remaining were matched at the block-face level.

Spatial clustering of supermarkets and fast food restaurants was determined using a spatial scan statistic performed with the software SaTScan (Martin Kulldorff, Boston, Mass) (28,29). The statistic uses a circular window of variable radius that moves across the map. The null hypothesis was that the rate of fast food restaurants or supermarkets (the number of businesses expected per 100,000 population) was the same in all windows. Clusters were defined as areas that had either a lower or higher rate of fast food restaurants or supermarkets than expected. The process of cluster detection was run through 999 Monte Carlo permutations of the data set to identify combinations of clusters of higher and lower rates of fast food restaurants (or supermarkets). The analyses were purely spatial, with a maximum cluster size of 50% of the population size. A Poisson distribution was assumed. The output provided the most likely cluster as well as several secondary clusters. Data associated with these clusters included the location identifiers, search radius and center coordinates, standard rate ratio, and a *P* value based on the log likelihood ratio test for each cluster. The cluster results were mapped in ArcGIS version 9 (ESRI, Redlands, Calif).

The SaTScan method was run eight times using different data and parameters. We assessed separately the spatial clustering of supermarkets and fast food restaurants regardless of the audit results. Next, we adjusted for the underlying racial distribution and poverty rate based on 2000 census data and determined whether the areas of higher or lower than expected number of supermarkets were still present. We then examined spatial clustering of supermarkets and fast food restaurants that scored in the highest tertile. We also adjusted these results by the racial distribution and poverty rate to determine whether these factors could explain the spatial clustering.

## Results

Table 1 provides a summary of the distribution of race and poverty within the 220 census tracts sampled as well as the distribution of supermarkets and fast food restaurants. Eighty-four tracts had less than 10% of the population living in poverty. Of these 84 tracts, 72 tracts were primarily white, 12 tracts were racially mixed, and none was primarily African American. In contrast, of the 83 census tracts in which 20% or more of the population was living in poverty, two tracts were primarily white, and 47 tracts were primarily African American.

Table 1 also provides information on the number of supermarkets and fast food restaurants ranked in the highest tertile within each of these areas. Eighty-one supermarkets and 355 fast food restaurants were identified in the study area. Of the 26 supermarkets in the highest tertile, 22 were in primarily white census tracts, and none were in primarily African American census tracts, regardless of poverty rate. Of the 120 fast food restaurants in the highest tertile, 63 were in primarily white census tracts, 53 were in racially mixed census tracts, and four were in primarily African American census tracts. There were fewer supermarkets and fast food restaurants that provided the best opportunity to meet recommended intake in primarily African American communities than in primarily white communities.

Next we ascertained whether differences in the distribution of supermarket and fast food restaurants were significantly different from what would be expected by chance alone, taking into account population density.

### Supermarket clustering

**Figure 1:**
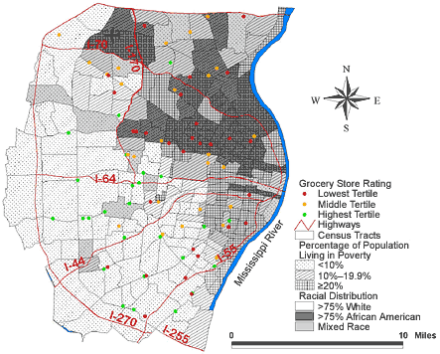
Location of 81 supermarkets and 220 census tracts with underlying racial distribution and poverty rates in the St Louis, Mo, study area.

Figure 1 shows the location of the 81 supermarkets in the study area; the area has a rate of 8.9 supermarkets per 100,000 population. Table 2 identifies the number of possible clusters of supermarkets within the study area. The unadjusted analysis showed no clustering of supermarkets; the most likely cluster showed *P* = .86. Adjusting for racial distribution and poverty rate resulted in similar findings: there was no clustering of supermarkets detected in the study area; the most likely cluster showed *P* = .48.

**Figure 2:**
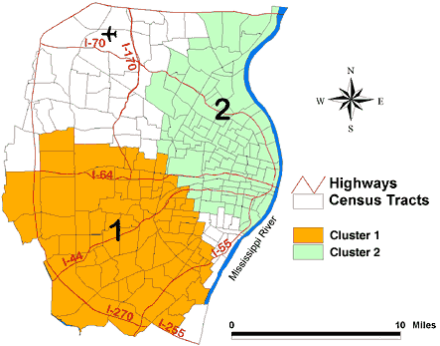
Unadjusted geographic clustering of supermarkets in the highest tertile, indicating greatest selection of healthy food markets in the St Louis, Mo, study area. The ratio of observed to expected number of supermarkets in Cluster 1 is 2.4 (*P* = .001); in Cluster 2, 0.0 (*P* = .003).

Of the 81 supermarkets, 26 were in the highest tertile. Table 2 shows two clusters of supermarkets in the highest tertile, and Figure 2 shows their location. Cluster 1, located in the southern part of the study area, had 23 supermarkets in the highest tertile when only 9.7 had been expected (ratio of observed/expected = 2.4; *P* = .001). This area included census tracts that were primarily white or racially mixed.

Alternately, Cluster 2 was located in the northeastern part of the study area (Figure 2). Nine supermarkets in the highest tertile were expected to be found, but there were no supermarkets in this area (ratio of observed/expected = 0, *P* = .003). The 311,491 primarily African American and lower-income people in this area were less likely than expected to have access to supermarkets where healthy choices were available.

Adjusting for the underlying racial distribution and poverty rate for the supermarkets in the highest tertile resulted in no significant clusters (*P* = .11).

### Fast food restaurant clustering 

**Figure 3:**
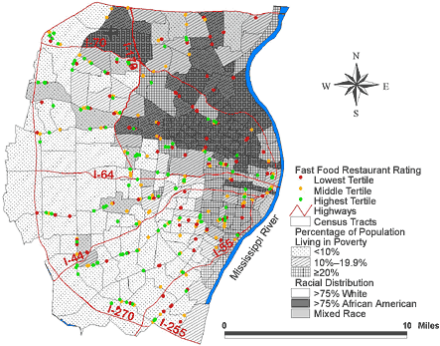
Location of 355 fast food restaurants and 220 census tracts with underlying racial distribution and poverty rate in the St Louis, Mo, study area.

**Figure 4:**
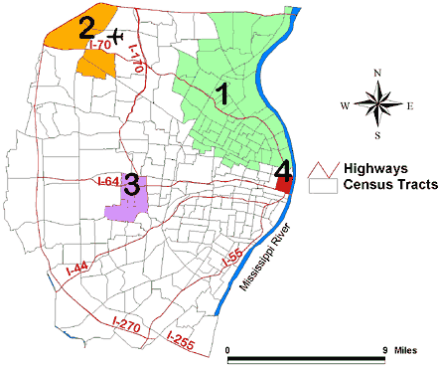
Unadjusted geographic clustering of fast food restaurants in the St Louis, Mo, study area. The ratio of observed to expected number of restaurants in Cluster 1 is 0.4 (*P* = .001); Cluster 2, 3.4 (*P* = .001); Cluster 3, 3.2 (*P* = .02); Cluster 4, 12.0 (*P* = .03).

There were 355 fast food restaurants located in the study area with a rate of 39.0 restaurants per 100,000 population (Figure 3).  Table 3 shows that four clusters were identified in the unadjusted analysis; Figure 4 shows their location. In Cluster 1, representing 52 census tracts and 20.6% of the study area population, 73 fast food restaurants were expected, and 31 were observed (ratio of observed/expected = 0.4; *P* = .001). The other three clusters indicated a higher than expected number of fast food restaurants but were based on a much smaller number of census tracts.

**Figure 5:**
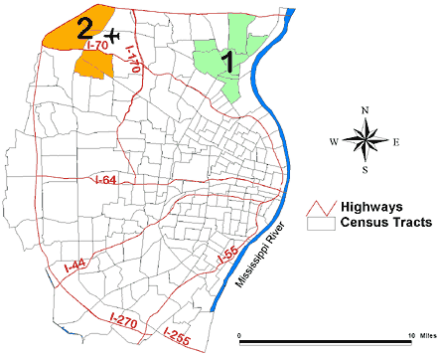
Geographic clustering of fast food restaurants adjusted for racial distribution and poverty rate by census tract in the St Louis, Mo, study area. The ratio of observed to expected number of restaurants in Cluster 1 is 0.07 (*P* = .004); Cluster 2, 3.1 (*P* = .001).

After adjusting for the racial distribution and poverty rate, only two clusters remained (Figure 5). Cluster 1 still showed fewer fast food restaurants than expected (ratio of observed/expected = 0.1; *P* = .004), but the area of Cluster 1 in the adjusted analysis contained only eight census tracts (Table 3). Cluster 2 remained unchanged in size and location after adjustment for racial distribution and poverty rate (ratio of observed/expected = 3.1; *P* = .001). Cluster 3 in the unadjusted analysis was no longer statistically significant after adjustment (*P* = .69). In addition, Cluster 4 in the unadjusted analysis was also no longer significant after adjustment (*P* = .053).

**Figure 6:**
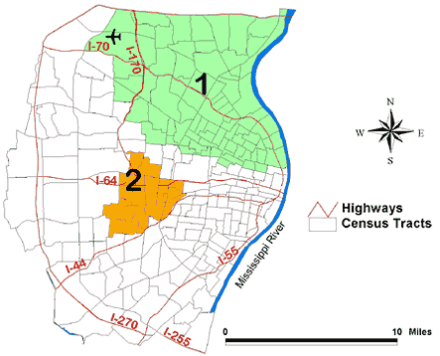
Unadjusted geographic clustering of fast food restaurants in highest tertile, indicating greatest selection of healthy food options in the St Louis, Mo, study area. The ratio of observed to expected number of restaurants in Cluster 1 is 0.3 (*P* = .001); Cluster 2, 3.0 (*P *= .01).

Of the 355 fast food restaurants, 120 were considered to be in the highest tertile. Two clusters were detected in the unadjusted analysis (Figure 6). Cluster 1 was located in the northern part of the study area and consisted of 75 census tracts and 31.7% of the study area population. The people in this cluster, primarily African American and lower income, were less likely than expected to have fast food restaurants in the highest tertile (ratio of observed/expected = 0.3; *P* = .001). A second, much smaller cluster was located mainly in the east central part of St Louis County and consisted of 17 census tracts. People in this cluster, primarily white and middle to higher income, were more likely than expected to have access to fast food restaurants that offered healthier food options (ratio of observed/expected = 3.0; *P* = .01). After adjustment for racial distribution and poverty rate, no clusters were detected.

## Discussion

Over the past several years researchers have made progress in assessing the role that the food environment plays in eating patterns. Previous work has shown a positive correlation between consumption of fruits and vegetables and the location of grocery stores (17,18). Others have found a positive association between what people eat and the selection of healthy options in the supermarket (20,24). We have also increased our understanding of the neighborhood factors that influence or are associated with differences in the food environment. Researchers have found better access to supermarkets in wealthier communities than in poorer communities and in white neighborhoods than in African American neighborhoods (17,18). More recent work indicates, however, that it may not be the location of the food outlets but the selection of food in the outlets that is associated with the ability to meet recommendations on dietary intake (30).

The primary purpose of this study was to determine whether there were differences in the extent to which populations have access to the infrastructures — fast food restaurants and supermarkets — necessary to adopt the eating behaviors recommended by the USDA to reduce chronic disease and promote health. Our work expanded the inquiry into access to foods that meet dietary recommendations and neighborhood characteristics. Similar to findings in other studies (18,19), our results showed that there were differences among neighborhoods in the location of food outlets. Our results also showed differences in the  availability of healthy food options. Moreover, our data suggest that the differences are at least partially explained by differences in racial distribution and poverty rates. These two factors (race and income) seem to be associated not only with the location of food outlets but also with the selection of food available that enables individuals to follow dietary recommendations (as seen in the analysis of the supermarkets and fast food restaurants in the highest tertile). The data suggest that individuals living in mixed or white high-poverty areas and in primarily African American areas (regardless of income) are less likely to have access to food outlets than individuals in primarily white, higher-income communities. Also, the food available in mixed or white high-poverty areas and in primarily African American areas is less likely to enable individuals to make healthy choices than food available in primarily white, higher-income communities.

This study has several limitations. First, our study is limited to an urban midwestern region, and the findings may be different in other areas nationally and internationally. Second, our study is limited to an area that has a primarily African American and white population. The relationships of interest may be different when making comparisons among racial and ethnic minority communities and between these communities and white communities. In addition, people may not necessarily eat where they live. Like other researchers, we examined the number of food outlets within a census tract (18) rather than the distance from a neighborhood center to the food outlet (19). We made a similar assumption: although it is possible that individuals and groups leave their geographic area to find healthy food options, many individuals, particularly those in lower-income communities, do not have access to cars or public transportation to allow this type of movement regularly. Lack of transportation was likely among residents living in our study area; according to the 2000 census, almost 50% of residents (48.8%) do not have a vehicle available (25). Thus, the location of food outlets within a census tract seems to be a reasonable indicator of access when conducting an area-level analysis. Another limitation to our work is that it is possible that the location of food outlets is a function of other geographic factors, such as proximity to a highway, mall, or airport. It might be useful for future studies to examine the potential influence of these other geographic factors and land-use patterns on food access.

Lastly, there is a compelling and rational economic argument that supermarkets and restaurants do not sell items that will not be purchased. Therefore, our findings (differential access to recommended food options) may be the result of behavior rather than the cause of behavior. It is impossible from a cross-sectional study such as ours to determine causality. The purpose of our study was to determine whether there were differences in the extent to which populations have access to the infrastructures necessary to adopt the eating behaviors recommended by the USDA to reduce chronic disease and promote health. Although we found differences according to racial composition and poverty level, our work does not indicate why these differences exist. Moreover, our work does not incorporate many of the other factors that influence dietary habits or purchasing (e.g., individual knowledge and skills, household size and composition, cultural factors). Future studies, both qualitative and quantitative, would assist in furthering our understanding of these issues.

Regardless of the reasons, some communities have less access than others to the food necessary for meeting recommended eating behaviors. Without a change in access to these foods, individuals cannot change their eating behaviors. If indeed these eating patterns are required to reduce chronic disease and promote health, then these communities will continue to have disparities in critical health outcomes unless we work to change current conditions. We in public health must begin to work collaboratively with our business communities and political structures to make it reasonable, rational, and economically sound to provide equal access to healthy choices.

## Figures and Tables

**Table 1:** Access to Supermarkets and Fast Food Restaurants by Racial Distribution and Level of Poverty Among 220 Census Tracts, St Louis, Mo

**Racial Composition and Poverty Level**	**Total Population**	**No. of Census Tracts**	**No. of Supermarkets**	**No. of Supermarkets in Highest Tertilea[Table-fn T1FN1]**	**No. of Fast Food Restaurants**	**No. of Fast Food Restaurants in Highest Tertilea[Table-fn T1FN1]**
**All census tracts sampled**	904,110	220	81	26	355	120
**<10% of population lives in poverty**	392,062	84	36	19	170	72
≥75% white	344,066	72	30	17	123	50
≥75% African American	0	0	0	0	0	0
Mixed	47,996	12	6	2	47	22
**10%–19.9% of population lives in poverty**	251,040	53	28	6	102	25
≥75% white	76,535	18	10	4	43	13
≥75% African American	74,082	13	11	0	15	1
Mixed	100,423	22	7	2	44	11
**≥20%** **of population lives in poverty**	261,008	83	10	0	28	3
≥75% white	6,646	2	1	1	0	0
≥75% African American	130,872	47	10	0	28	3
Mixed	123,490	34	6	0	55	20

a Each restaurant and supermarket was assigned a rating of high, medium, or low potential for meeting dietary intake recommendations as established by the U.S. Department of Agriculture (27).

**Table 2:** Spatial Clustering of Supermarkets Within 220 Census Tracts, St Louis, Mo

**Possible Cluster Identifed**	**Observed No. of Supermarkets**	**Expected No. of Supermarkets**	**Ratio of Observed to Expected No. of Supermarkets**	**Population in Cluster (No. of Census Tracts)**	**Log Likelihood Ratio (*P*)**
**All supermarkets (unadjusted)**					
1	41	27.8	1.5	309,874 (77)	4.55 (.86)
**All supermarkets (adjusted for racial composition and poverty rate)**					
1	54	38.9	1.4	456,825 (113)	5.69 (.48)
**Supermarkets in highest tertile^a^ (unadjusted)**					
1	23	9.7	2.4	335,664 (76)	14.88 (.001)
2	0	9.0	0.0	311,491 (96)	10.98 (.003)
**Supermarkets in highest tertile^a^ (adjusted for racial composition and poverty rate)**					
1	9	2.3	4.0	46,531 (10)	6.80 (.11)

a Each restaurant and supermarket was assigned a rating of high, medium, or low potential for meeting dietary intake recommendations as established by the U.S. Department of Agriculture (27).

**Table 3:** Spatial Clustering of Fast Food Restaurants Within 220 Census Tracts, St Louis, Mo

**Possible Cluster Identified**	**Observed No. of Fast Food Restaurants**	**Expected No. of Fast Food Restaurants**	**Ratio of Observed to Expected No. of Fast Food Restaurants**	**Population in Cluster (No. of Census Tracts)**		**Log Likelihood Ratio (*P*)**
**All fast food restaurants (unadjusted)**						
1	31	73.3	0.4	187,873 (52)		18.65 (.001)
2	30	8.8	3.4	22,432 (4)		16.37 (.001)
3	20	6.3	3.2	16,052 (4)		9.76 (.02)
4	6	0.5	12.0	1,280 (1)		9.46 (.03)
**All fast food restaurants (adjusted for racial composition and poverty rate)**						
1	1	15.0	0.1	42,378 (8)		11.56 (.004)
2	30	9.8	3.1	22,432 (4)		13.97 (.001)
3	20	8.9	2.6	16,052 (4)		5.33 (.69)
4	6	0.6	10.7	1,280 (1)		8.82 (.053)
**Fast food restaurants in highest tertile^a^ (unadjusted)**						
1	11	38.0	0.3	289,181 (75)		17.43 (.001)
2	23	7.7	3.0	58,512 (17)		10.97 (.01)
**Fast food restaurants in highest tertile^a^ (adjusted for racial composition and poverty rate)**						
0	0	7.0	0.0	105,184 (22)	7.20 (.18)	

a Each restaurant and supermarket was assigned a rating of high, medium, or low potential for meeting dietary intake recommendations as established by the U.S. Department of Agriculture (27).
